# Initial motor skill performance predicts future performance, but not learning

**DOI:** 10.1038/s41598-023-38231-5

**Published:** 2023-07-13

**Authors:** Dekel Abeles, Jasmine Herszage, Moni Shahar, Nitzan Censor

**Affiliations:** 1 School of Psychological Sciences, Tel Aviv University, 69978 Tel Aviv, Israel; 2 Sagol School of Neuroscience, Tel Aviv University, 69978 Tel Aviv, Israel; 3 AI and Data Science Center of Tel Aviv University (TAD), 69978 Tel Aviv, Israel

**Keywords:** Human behaviour, Learning and memory

## Abstract

People show vast variability in skill performance and learning. What determines a person's individual performance and learning ability? In this study we explored the possibility to predict participants’ future performance and learning, based on their behavior during initial skill acquisition. We recruited a large online multi-session sample of participants performing a sequential tapping skill learning task. We used machine learning to predict future performance and learning from raw data acquired during initial skill acquisition, and from engineered features calculated from the raw data. Strong correlations were observed between initial and final performance, and individual learning was not predicted. While canonical experimental tasks developed and selected to detect average effects may constrain insights regarding individual variability, development of novel tasks may shed light on the underlying mechanism of individual skill learning, relevant for real-life scenarios.

## Introduction

People vary substantially in their ability to execute daily skills. To determine the sources of such variability, most studies have focused on initial and online task performance, known to vary between individuals^1^. Thus, with no prior practice, some individuals might exhibit outstanding performance, while others might express slow and inaccurate performance. Importantly, people vary greatly in their ability to learn new skills as well, with the range of possible improvement differing between individuals. Predicting performance and learning based on early skill acquisition offers an abundance of benefits and may be useful for effective adjustment of training regimes in daily life and for neurorehabilitation. What determines individual differences in performance and learning abilities? Here, we aimed to investigate individual differences in skill learning by predicting future performance and the amount of learning an individual will exhibit across different time intervals, based on information extracted from performance at an early session.

Investigating individual differences with complex statistical modeling requires a large pool of participants. Therefore to address this question, we leveraged online platforms enabling crowdsourced recruitment, producing large-scale data sets^2,3,4^.

To predict the extent of learning from skill acquisition characteristics, we utilized a common motor sequence learning task^5^, requiring participants to perform a sequence of finger movements as fast and as accurate as possible. This motor sequence learning task is widely used to model human skill acquisition and learning in health and disease^6–18^. To focus on procedural learning in the task, and control for semantic properties of the sequence itself, the required sequence is presented on the screen throughout the task. As evident in other motor learning tasks, this task is characterized by typical within-session learning curves, showing higher improvements at the beginning of a session and then reaching a plateau at the end of that session^8,19,20^. Importantly, practicing the task repeatedly during multiple days is known to induce improvements between-sessions, in the absence of additional practice (for example see^20^), known as ‘offline gains’, possibly depending on offline consolidation processes. In explicit motor learning this stage of learning is sleep-dependent, while such learning in implicit motor skill tasks has been shown to be time-dependent^11,21^. In general, these timelines showing incremental improvement in performance across days of practice are common across motor learning domains, similarly reported in visually guided curved movement^22^ and bimanual tasks^23,24^. Thereafter, if no additional practice is performed, learning gains might decrease over time due to memory decay. However, motor skills were previously shown to remain stable even after long periods of time without any additional practice of the task^25^. Of note, additional motor learning tasks such as force-field or visuomotor adaptation tasks commonly exhibit different dynamics, for a review on these and different motor learning domains see^26^.

To collect sufficient amount of data, we conducted a large-scale crowdsourced experiment, recruiting online participants to take part in 3 learning sessions, with a retention session following one week, and an additional long-term retention session following 2–4 months. First, we validated that online participation demonstrates common learning rates within each session as well as between sessions offline gains^5,11,27^. Next, we applied machine learning models based on engineered features derived from existing literature of motor skill, to predict future learning based on initial performance. Among the extracted features are common quantifications of task performance such as number of correct sequences, and response times, along with custom features designed to capture variable learning dynamics and the fitted parameters of the learning curve (learning rate and fatigue rates parameters).

## Methods

### Participants

Participants were recruited online from the Amazon Mechanical Turk platform (https://www.mturk.com). Qualifications for registered MTurk workers to participate in the first session of the experiment were: above 95% approval rate in previous MTurk assignments, currently located in the USA, right-handed, and did not previously participate in a sequential tapping task from our lab. Each of the following sessions were made available to qualified participants according to the predefined scheduling scheme and was available for 12 h. The age of participants ranged from 18 to 78 (see below). Data were collected using non overlapping batches of participants—session 1 of the experiment was made available on a Monday and the next sessions accordingly. This resulted in the following number of participants per session: Session 1: 571 participants, Session 2: 334, Session 3: 273, Session 4: 195, Session 5: 103. Additional exclusion criteria were enforced to make sure the remaining sample of participants were all attentive and complied with instructions (see below). This resulted in the final sample of: session 1: N = 460; 274 Female; Mean age = 43.35, Std = 12.99; session 2: N = 254; 154 Female; Mean age = 43.29, Std = 12.83; session 3: N = 203; 116 Female; Mean age = 44.07, Std = 12.72; session 4: N = 134; 75 Female; Mean age = 46.08, Std = 13.00; session 5: N = 75; 39 Female; Mean age = 47.48, Std = 12.47. Additionally collected participant information was not varied enough to be used as predictive features (Highest level of attained education: High-School = 26%, Bachelors = 46%, Masters = 17%; Below 2 h of weekly musical engagement: 82%; Below 2 h of weekly physical activity: 75%). All participants used a button press to sign an online informed consent form presented at the beginning of each session. The payment scheme for all sessions was visible on the experiment page on the Mturk platform. To minimize dropouts, the compensation increased as sessions progressed (1.5$, 2$, 2.5$, 2$ for the shorter 4th Retention session, and 5$ for the final long-term Retention session). The methods were performed in accordance with relevant guidelines and regulations and approved by Tel Aviv University’s Ethics committee.

### Task

Participants performed a procedural motor task—the sequence tapping task^5^, a canonical task used in numerous motor learning studies^20,28–30^. Participants were instructed (using illustrative slides) to place their non-dominant left hand on their keyboard in a one-to-one correspondence between fingers and digit-numbers; pinky—#1, ring finger—#2, middle finger—#3, index finger—#4. They were instructed to repeatedly tap the requested pattern (4-1-3-2-4) as fast and as accurate as possible using their left hand for the entire trial duration (10 s), according to accepted guidelines of the task^5,8^. A 10 s count-down screen preceded each trial and served as a break. Throughout each trial, each key press produced a dot displayed at the top portion of the screen, with the dots accumulating from left to right as the trial progressed^19,20,25^. Except for the sequence itself, this was the only visible item on the screen during the trial. The experiment was programed in Psychopy^31^ and was hosted on Pavlovia servers (https://pavlovia.org/).

### Experimental procedure

**Figure 1:**
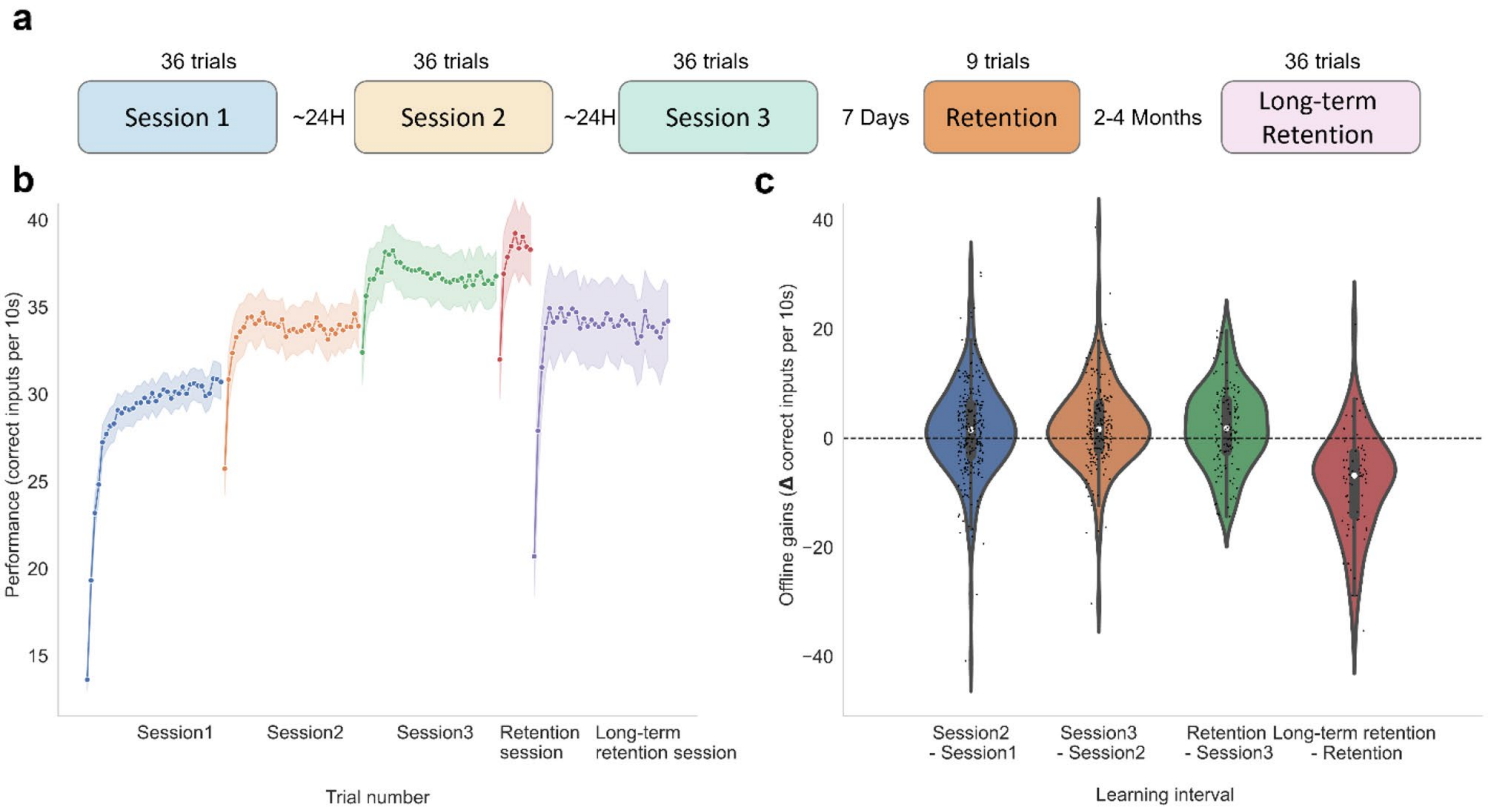
Task performance within and between sessions. (**a**) Experimental design. (**b**) Learning curves across all five sessions (session 1—blue, session 2—yellow, session 3—green, Retention session—orange, Long Term Retention session—pink), the shaded area represents the 95% confidence interval. (**c**) Offline gains between consecutive sessions. Data points in the violin plots represent offline gains for each participant. The white dot represents the median.

Before the first session, participants reported their age, gender, education level, time of weekly engagement with musical instruments and time engaged in physical activities. Additionally, at the beginning of each session, participants were asked to report the duration and the quality of sleep on the night preceding that session. At the end of each session, as a simple attention check, participants were asked to report the hand they used to perform the task (they were instructed to use their left hand in the beginning of the experiment). The study initially comprised of 4 sessions—each consisting of 36 trials except for the Retention session (4th session) containing 9 trials. A fifth session, the long-term Retention session, was made available 2–4 months after the completion of the Retention session, and comprised of 36 trials, identical to the first 3 sessions (Fig. 1a). The first three sessions were chosen to be 24 h apart to enable overnight consolidation between sessions^7,8,27^. The retention interval was chosen to be significantly longer (7 days) to examine skill retention absent of daily practice. The long-term retention interval was chosen to be very long (> 2 month) to examine skill deterioration effects stemming from memory decay.

### Data analysis

All analyses were performed using custom code written in python^32^. Data preprocessing and handling was done using the Numpy^33^ and Pandas^34^ package. The machine learning pipeline was defined using Scikit-learn^35^ and Pytorch^36^. The Matplotlib^37^ and Seaborn^38^ libraries were used for data visualization. Statistical analysis was conducted using Pingouin^39^.

Participants were qualified to continue to the next session if they did not end the experiment mid-session and averaged at least 9 input characters per trial. Additionally, to validate participants’ attention to the task, data were discarded from all sessions if participants were too slow to start the trial following a break (first input exceeded 2 s) or failed to respond in more than 5 trials per session. Next, if the reported sleep duration was outside of the acceptable range of 6–12 h, the data from that session and all following sessions were discarded.

Performance was defined as the overall number of correct keypresses in a trial^20,40–42^. To calculate the amount of correct keypress, the number of complete (4–1-3-2-4) sequences in a trial was multiplied by the sequence’s length (5). If the trial ended mid-pattern (e.g., 4-1-3), all keypresses from the start of that pattern were also considered correct. Between–session learning was quantified based on the maximal performance (the average of the 3 best trials; see Supplementary table s1).

### Machine learning modeling

To test the predictive power of the behavior observed during initial training (session 1) on future learning induced by subsequent training sessions, three-time intervals were examined: (a) change in performance from the 1st session to the 2nd session, (b) change in performance from the 1st session to the 3rd session, (c) change in performance from the 1st session to the 4th retention session. Two additional time intervals were used to predict skill retention (a) one week retention interval (from the 3rd session to the 4th) and (b) a long-term retention interval (2–4 months) (from the 4th session to the 5th).

#### Engineered features

Based on previous research in the field, and specifically on the sequential tapping task, engineered features were calculated from the behavioral data aimed to capture more complex patterns in the data. All extracted features and their definitions are reported in Supplementary table s1.

#### Model selection and training

The gradient boosting method on random forests^43^ was used due to its efficiency in tabular data prediction, and ability to extract complicated non-linear features without specifically designing them^44^. Due to the high number of potential measures in relation to participants for each prediction interval, a hyperparameter grid search was conducted, allowing for high values of regularization parameters to avoid potential overfitting to the training set (the best parameters were chosen based on a fivefold cross validation average MSE score). Additionally, to allow for a better feature selection process that will help with model interpretability, we exposed the model to an increasing set of potential predictors based on their complexity, starting with high level features (i.e., session dynamic parameters) and ending with the simplest features (performance per trial). Initially, only non-behavioral features were included (i.e., Age and Gender). Predictors were introduced in steps (see Supplementary table s1). In the 1st step parameters from the learning curve were introduced. The 2nd step included the parameters extracted to capture *Within sequence consistency dynamics* and the *pattern consistency trend*. The 3rd step included *Lowess based features*. The 4th step included *session statistics*. The 5th step included the micro-offline and micro-online features of the first 5 trials^19^. And the 6th and final step, included the performance per trial for all trials in the session. Note that hyperparameters were optimized specifically for each step due to the increased model complexity that comes with additional parameters and the need to adjust the regularization terms accordingly. Due to the large number of potential predictors, and as an additional means to avoid over-fitting to the training set, we tested these pipelines both with and without an additional preprocessing step of principle components analysis (PCA)-based dimensionality reduction. Data were first transformed into Z-scores before entering the model. For prediction purposes, the training set mean, and standard deviations were used.

To test whether the results were due to the specific modeling family of choice, different alternative models were examined and are described in the Supplementary materials.

#### Model evaluation

The parameters that resulted in the best performance on the training-set for each model type and prediction interval were used to re-train the model on the entire training set and examine it on the 20% of hold-out data that was not accessible during training. The final score is thus the reported explained variance (R^2^) of the hold-out dataset.

### Statistical analysis

One sample *t*-test was used to examine the statistical significance of the offline gains analysis. Correlational analyses were conducted using Pearson or Spearman correlations.

## Results

We first validated that performance was consistent with previous studies employing the same task in laboratory settings^8,25,41,42^. Indeed, participants displayed typical learning curves (Fig. 1b), with significant learning expressed both within-session, and between-sessions as offline gains^8,45,46^ (Fig. 1c). Specifically, there were significant offline gains between sessions 1 and 2 (*t*(253) = 2.639, *p* = 0.009, *Cohen’s d* = 0.126, *CI* = [0.36, 2.45]), and between sessions 2 and 3 (*t*(202) = 4.008, *p* < 0.001, *Cohen’s d* = 0.191, *CI* = [1.08, 3.16]). Interestingly, even when the skill memory was tested following one week, additional offline gains were evident, with a significant improvement between session 3 and Retention session 4 (*t*(133) = 3.154, *p* = 0.002, *Cohen’s d* = 0.183, *CI* = [0.75, 3.28]). In addition, during the long term retention interval, lasting between 2–4 months (see “Methods”) a significant reduction in performance was observed (difference from Retention (4th session) to Long-term Retention (5th session): *t*(74) = − 7.661, *p* < 0.001, *Cohen’s d* = 0.722, *CI* = [− 10.32, − 6.06]), indicating a decay of the memory trace over a period of months. Overall, these results validate typical within and between session motor skill learning.

**Figure 2:**
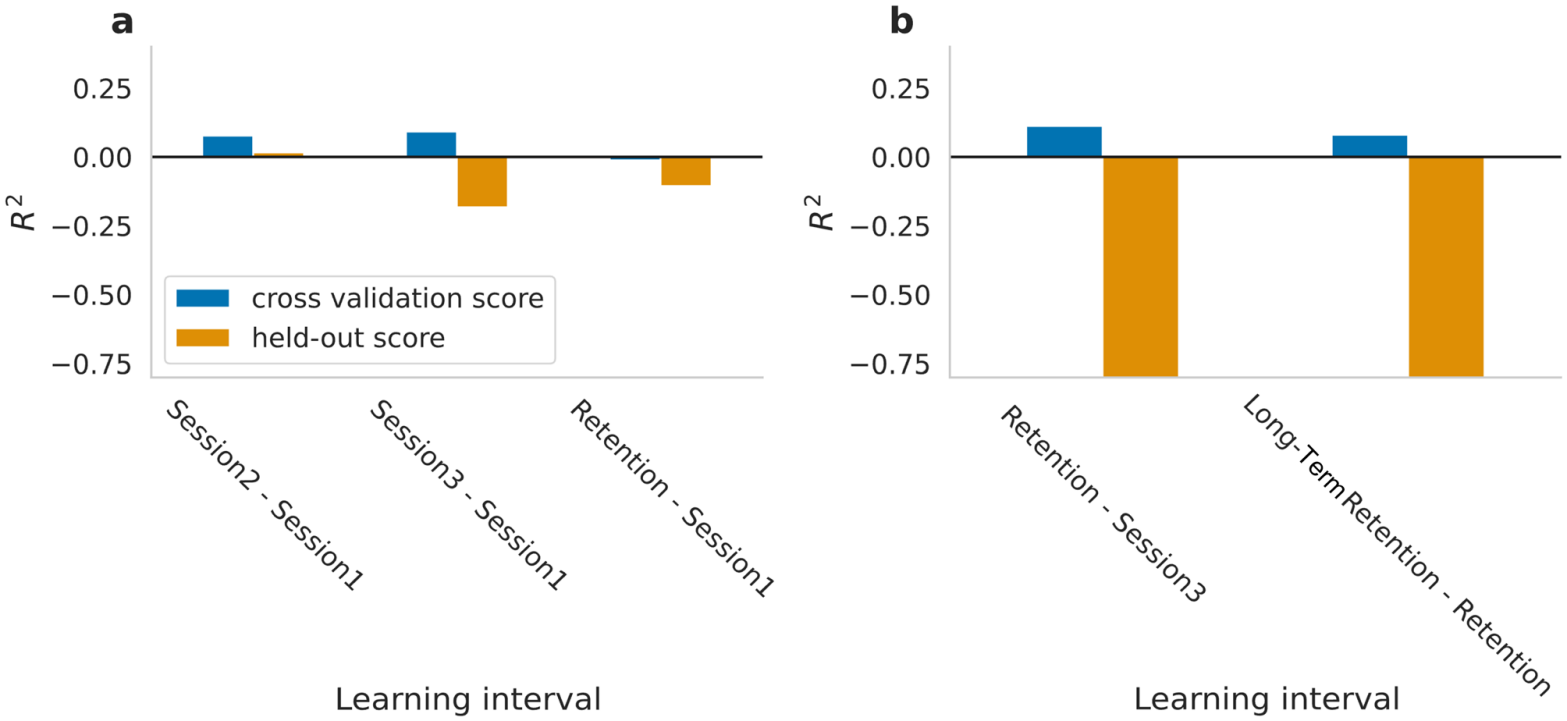
Model performance with engineered features. (**a**) Maximum mean cross-validation R^2^ scores (blue) and the corresponding hold-out R^2^ scores (orange) for each learning interval (x axis). (**b**) Maximum mean cross-validation R^2^ scores (blue) and the corresponding hold out R^2^ (orange) for the two retention intervals (x axis).

ML was then used to predict learning based on performance in the first session. To that end, our goal was to predict the improvements between performance in session 1 and performance in each of the subsequent sessions 2–4. To minimize within session effects of warm-up and fatigue^29,47^, between-session learning was quantified based on the maximal performance in each session (see “Methods”). Predictors were introduced in steps with diminishing feature complexity, ranging from whole session dynamics descriptors to the number of correct keypresses in each trial. The models did not predict learning in the hold-out set (*session2–session1*: *R*^*2*^_*mean_cv_score*_ = 0.08, *R*^*2*^_*test*_ = 0.016; *session3–session1*: *R*^*2*^_*mean_cv_score*_ = 0.09, *R*^*2*^_*test*_ = *−* *0.18; Retention session 4—session1: R*^*2*^_*mean_cv_score*_ = − 0.01, *R*^*2*^_*test*_ = − 0.1, Fig. 2a). Of note, a negative R^2^ score indicates that model predictions do not explain any variance in the dependent variable.

Is behavior at initial stages of skill acquisition indicative of skill retention? To address this question, the model was trained to predict the performance change during the short (from session 3 to Retention session) and long retention intervals (from Retention to Long-term retention), based on performance in either the first or all 3 prior sessions. The change in performance over both retention intervals was not predicted by the best performing model (highest cross validation score) as reflected in the negative *R*^*2*^ in the hold-out set (*Retention session—session3*: *R*^*2*^_*mean_cv_score*_ = 0.11, *R*^*2*^_*test*_ = − 0.84; *Long-retention—Retention session: R*^*2*^_*mean_cv_score*_ = 0.08 *R*^*2*^_*test*_ = − 0.88*,* Fig. 2b)*.* Since the long-term retention interval showed negative changes in performance, further investigation of the data revealed that maximum performance in the Retention session was the best predictor for the subsequent long-term retention interval (Pearson’s *r*(73) = − 0.49, *p* < 0.001, *CI* = [− 0.65, − 0.30]). Considering that maximum performance in the Retention session reflects both innate abilities and the overall benefit of training throughout the experiment, we examined the correlation between total learning and retention. Pearson correlation confirmed that the amount of total learning throughout the experiment (performance differences between session 1 and Retention session 4) was even a stronger predictor of the change in performance (Pearson’s *r*(73) = − 0.58, p < 0.001, *CI* = [− 0.71, − 0.40]), suggesting that participants exhibit long-term decay of their own learning before the retention interval. These results were not due to a specific modeling family of choice and were consistent when approaches with different inductive biases were used, including a convolutional network that benefits from the temporal relation between adjunct datapoints or a LSTM (long short-term memory) network that is specifically suitable for time-series (see Supplementary materials).

**Figure 3:**
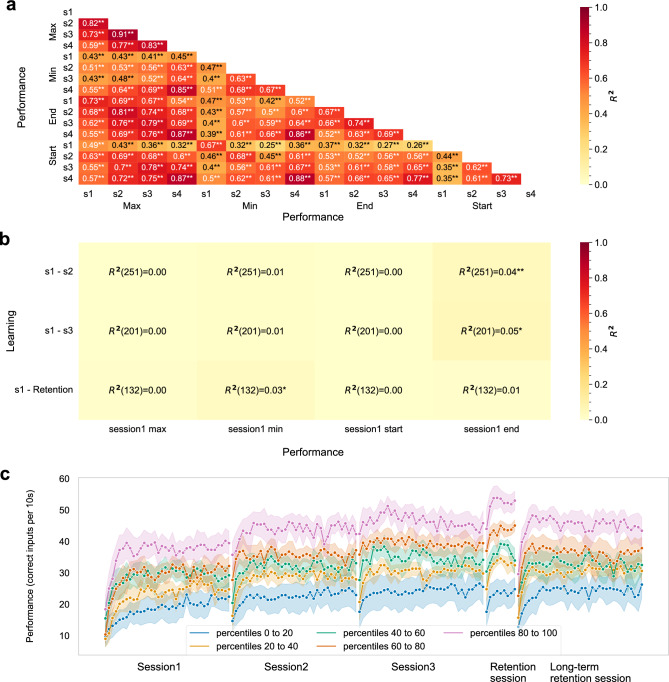
Performance was consistent across sessions but did not predict learning. (**a**) Performance in all sessions explains a large portion of the variability in future performance (*R*^*2*^ range = [0.25, 0.91]. (**b**) Performance hardly explains the variability in learning (*R*^*2*^ range = [0, 0.05]. (**c**) Performance throughout the experiment separated according to the performance quantile in the Retention session (colors), showing that participants' relative performance rank remains stable across sessions. Shaded areas represent the 95% confidence interval. Statistical significance is marked with * for p < 0.05 and with ** for p < 0.001.

To further investigate the above results, we assessed the consistency of simple performance metrics in each session and between-session learning, using Pearson correlations. Performance in each session explained a large portion of the variance in Performance scores across the 3 sessions and Retention session (R^2^ range = [0.25–0.91], all p < 0.001; see Fig. 3a), indicating high test–retest reliability and thus a stable measure of individual performance. However, performance hardly explained any portion of the variance in learning (R^2^ range = [0.00, 0.05]; Fig. 3b). While these results suggest that variability in performance can be explained by performance in previous sessions, variability in learning can hardly be explained. To further illustrate this point, participants were separated into 5 quantile ranges (each spanning 20%)^48^ based on their maximum performance in the Retention session, plotted throughout the experiment (Fig. 3c). The plotted curves show that participant’s relative performance remained stable throughout the experiment.

## Discussion

The goal of this study was to identify what determines an individual's skill performance and learning ability, based on their initial behavior during skill acquisition. Learning was measured at different intervals, using large-scale crowdsourced data. Results showed improvements in performance throughout the first 3 sessions and the retention session following one week. Between session improvements without additional practice during retention intervals of several days^20,24,49^ may reflect enhanced offline consolidation mechanisms^50^, and is in line with between-days spacing effects^51^. Interestingly, performance in early sessions did not predict subsequent learning, even though variability in performance was explained by performance in previous sessions. In addition, participants exhibited long-term skill memory decay, bound by their own learning before the retention interval.

Machine learning was leveraged to predict learning. We extracted features from the observed behavior in the task, ranging from high level features such as the parameters of the learning curve (learning rate, fatigue accumulation rate), to simple features such as the correct number of keypresses in a trial. The model did not predict future learning, and the results were consistent when validated across different modeling approaches. Thus, no further interpretability attempts were taken. It is worth noting that when models are predictive, examining their decision functions could help form future hypotheses regarding the suspected cognitive meaning of successful predictors which could then be explicitly tested in future experiments.

It is conceivable that the learning measures employed are noisy or do not reflect a stable individual trait. Low test–retest reliability of individual metrics was demonstrated in other fields, such as the field of attentional control, where many canonical tasks, including Stroop^52^, Flanker^53^, and Navon^54^ result in robust between-conditions experimental effects, but in unreliable estimates of individual effects^55^, thus limiting insights regarding individual differences. Spearman and colleagues attributed this limitation to the calculation of a composite score as the difference between two measurements for the same individual^56,57^. Critically, such differences between two measurements are the key outcome for evaluating skill learning. Therefore, while skill learning tasks have extensively shown robust and replicable results across different contexts when examined between conditions^20,25,41,42,58^, insights into individual differences may be limited. Accordingly, while large sample sizes may reduce standard errors and enable to detect average between-conditions effects, they do not necessarily improve the reliability of individual effects. This issue could be addressed by increasing the number of repeated measures or trials for each participant, as done for example in studies of perceptual learning^59^.

Separating participants into sub-groups based on their performance in the Retention session resulted in a visible, consistent classification throughout all sessions, suggesting that future learning may be too small to change participants’ rank. Participants showing higher performance at the beginning also show better performance at the end of the experiment. These results are consistent with previous findings of a large online sample of participants playing a complex online shooter game^48^. When participants were split into 5 quantile ranges based on their best performance the curves remained separated from the very beginning of the task. Development of novel model motor skill tasks with high variability in between-session learning, and in which future performance is not determined by initial performance, may overcome the above constraints. Furthermore, the challenge of designing such novel tasks, would be to construct them as generalizable across different motor domains (including reaching models^22^ and bimanual tasks^23,24^), and across different contexts which are known to affect performance^60–62^. Such tasks may provide valuable insights regarding learning variability, which may be further combined with potentially useful predictors from other domains^1,63,64^, functional and anatomical neuroimaging information^65^, or high-resolution kinematic inputs^66^.

The age of participants is known to affect their motor performance and learning^67^. To address this issue, participants in the current study were recruited from a large variability of ages (see “Methods”), which may have supported more generalizable results. However, since all participants were adults, future studies should evaluate developmental differences in motor learning in children where performance dynamics and their association with future performance might be different. Additionally, while this study tested motor learning, results might be generalized to other procedural learning domains, such as perceptual learning, which is known to share common characteristics with procedural learning (for a review see^68^). Nevertheless, the generalization of the current results to other learning domains should be examined in future studies.

In correspondence with other empirical fields testing human behavior, canonical experimental tasks developed and selected to detect average effects may constrain insights regarding individual variability, relevant for real-life scenarios. Accordingly, development of novel tasks with high test–retest reliability which model real-life learning, may shed light on the underlying mechanisms of individual differences in skill learning and promote personalized learning regimes geared to enhance human performance.

## Supplementary Information

Supplementary Information.

## Data Availability

The datasets generated and analyzed during the current study are publicly available at https://osf.io/rxmpz.

## References

[1] Anderson DI, Lohse KR, Lopes TCV, Williams AM (2021). Individual differences in motor skill learning: Past, present and future. Hum. Mov. Sci..

[2] Chandler J, Shapiro D (2016). Conducting clinical research using crowdsourced convenience samples. Annu. Rev. Clin. Psychol..

[3] Ranard BL (2014). Crowdsourcing—Harnessing the masses to advance health and medicine, a systematic review. J. Gen. Intern. Med..

[4] Johnson BP, Dayan E, Censor N, Cohen LG (2021). Crowdsourcing in Cognitive and Systems Neuroscience. Neurosci..

[5] Karni A (1995). Functional MRI evidence for adult motor cortex plasticity during motor skill learning. Nature.

[6] Genzel L (2012). Complex motor sequence skills profit from sleep. Neuropsychobiology.

[7] Muellbacher W (2002). Early consolidation in human primary motor cortex 3. Nature.

[8] Karni A (1998). The acquisition of skilled motor performance: Fast and slow experience-driven changes in primary motor cortex. Proc. Natl. Acad. Sci. U.S.A..

[9] Cohen DA, Pascual-Leone A, Press DZ, Robertson EM (2005). Off-line learning of motor skill memory: A double dissociation of goal and movement. Proc. Natl. Acad. Sci. U.S.A..

[10] Reis J (2009). Noninvasive cortical stimulation enhances motor skill acquisition over multiple days through an effect on consolidation. Proc. Natl. Acad. Sci. U.S.A..

[11] Robertson EM, Pascual-Leone A, Press DZ (2004). Awareness modifies the skill-learning benefits of sleep. Curr. Biol..

[12] Wu J, Srinivasan R, Kaur A, Cramer SC (2014). Resting-state cortical connectivity predicts motor skill acquisition. Neuroimage.

[13] Wiestler T, Diedrichsen J (2013). Skill learning strengthens cortical representations of motor sequences. Elife.

[14] Brown RM, Robertson EM (2007). Inducing motor skill improvements with a declarative task. Nat. Neurosci..

[15] Perez MA (2007). Neural substrates of intermanual transfer of a newly acquired motor skill. Curr. Biol..

[16] Dayan E, Laor-Maayany R, Censor N (2016). Reward disrupts reactivated human skill memory. Sci. Rep..

[17] Gabitov E, Boutin A, Pinsard B, Censor N, Fogel SM, Albouy G, King BR, Carrier J, Cohen LG, Karni A, Doyon J (2019). Susceptibility of consolidated procedural memory to interference is independent of its active task-based retrieval. PLoS One..

[18] Censor N, Buch ER, Nader K, Cohen LG (2016). Altered Human Memory Modification in the Presence of Normal Consolidation. Cereb. Cortex.

[19] Bönstrup M (2019). A rapid form of offline consolidation in skill learning. Curr. Biol..

[20] Herszage J, Sharon H, Censor N (2021). Reactivation-induced motor skill learning. Proc. Natl. Acad. Sci. U.S.A..

[21] Robertson EM, Pascual-Leone A, Miall RC (2004). Current concepts in procedural consolidation. Nat. Rev. Neurosci..

[22] Shmuelof L, Krakauer JW, Mazzoni P (2012). How is a motor skill learned? Change and invariance at the levels of task success and trajectory control. J. Neurophysiol..

[23] Beets IAM (2012). Active versus passive training of a complex bimanual task: Is prescriptive proprioceptive information sufficient for inducing motor learning?. PLoS One.

[24] Haith AM, Yang CS, Pakpoor J, Kita K (2022). De novo motor learning of a bimanual control task over multiple days of practice. J. Neurophysiol..

[25] Herszage J, Censor N (2017). Memory reactivation enables long-term prevention of interference. Curr. Biol..

[26] Krakauer JW (2006). Motor learning: Its relevance to stroke recovery and neurorehabilitation. Curr. Opin. Neurol..

[27] Lugassy D, Herszage J, Pilo R, Brosh T, Censor N (2018). Consolidation of complex motor skill learning: Evidence for a delayed offline process. Sleep.

[28] Bönstrup M, Iturrate I, Hebart MN, Censor N, Cohen LG (2020). Mechanisms of offline motor learning at a microscale of seconds in large-scale crowdsourced data. npj Sci. Learn..

[29] Rickard TC, Cai DJ, Rieth CA, Jones J, Ard MC (2008). Sleep does not enhance motor sequence learning. Cognition.

[30] Albouy G (2012). Neural correlates of performance variability during motor sequence acquisition. Neuroimage.

[31] Peirce J (2019). PsychoPy2: Experiments in behavior made easy. Behav. Res. Methods.

[32] Van Rossum G, Drake FL (1995). Python Reference Manual.

[33] Harris CR (2020). Array programming with {NumPy}. Nature.

[34] McKinney W (2010). Data structures for statistical computing in Python. Proc. 9th Python Sci. Conf..

[35] Pedregosa F (2011). Scikit-learn: Machine learning in Python. J. Mach. Learn. Res..

[36] Paszke A (2019). PyTorch: An imperative style, high-performance deep learning library. Adv. Neural Inf. Process. Syst..

[37] Hunter JD (2007). Matplotlib: A 2D graphics environment. Comput. Sci. Eng..

[38] Waskom ML (2021). seaborn: Statistical data visualization. J. Open Source Softw..

[39] Vallat R (2018). Pingouin: Statistics in Python. J. Open Source Softw..

[40] Censor N, Horovitz SG, Cohen LG (2014). Interference with existing memories alters offline intrinsic functional brain connectivity. Neuron.

[41] de Beukelaar TT, Woolley DG, Wenderoth N (2014). Gone for 60 seconds: Reactivation length determines motor memory degradation during reconsolidation. Cortex.

[42] Korman M (2007). Daytime sleep condenses the time course of motor memory consolidation. Nat. Neurosci..

[43] Chen, T. & Guestrin, C. {XGBoost}: A scalable tree boosting system. In *Proceedings of the 22nd ACM SIGKDD International Conference on Knowledge Discovery and Data Mining* 785–794 (ACM, 2016). 10.1145/2939672.2939785

[44] Shwartz-Ziv R, Armon A (2022). Tabular data: Deep learning is not all you need. Inf. Fusion.

[45] Walker MP, Brakefield T, Morgan A, Hobson JA, Stickgold R (2002). Practice with sleep makes perfect: Sleep-dependent motor skill learning. Neuron.

[46] Press DZ, Casement MD, Pascual-Leone A, Robertson EM (2005). The time course of off-line motor sequence learning. Cogn. Brain Res..

[47] Adams JA (1952). Warm-up decrement in performance on the pursuit-rotor. Am. J. Psychol..

[48] Stafford T, Dewar M (2014). Tracing the trajectory of skill learning with a very large sample of online game players. Psychol. Sci..

[49] Atienza M, Cantero JL, Stickgold R (2004). Posttraining sleep enhances automaticity in perceptual discrimination. J. Cogn. Neurosci..

[50] Dudai Y, Karni A, Born J (2015). The consolidation and transformation of memory. Neuron.

[51] Stickgold R, James L, Hobson JA (2000). Visual discrimination learning requires sleep after training. Nat. Neurosci..

[52] Stroop JR (1935). Studies of interference in serial verbal reactions. J. Exp. Psychol..

[53] Eriksen B, Eriksen CW (1974). Effects of noise letters upon the identification of a target letter in a nonsearch task*. Percept. Psychophys..

[54] Navon D (1977). Forest before trees: The precedence of global features in visual perception. Cogn. Psychol..

[55] Hedge C, Powell G, Sumner P (2018). The reliability paradox: Why robust cognitive tasks do not produce reliable individual differences. Behav. Res. Methods.

[56] Cronbach LJ, Furby L (1970). How we should measure" change": Or should we?. Psychol. Bull..

[57] Spearman, C. The proof and measurement of association between two things (1961). 10.1093/ije/dyq191 21051364

[58] Gabitov E (2017). Re-stepping into the same river: Competition problem rather than a reconsolidation failure in an established motor skill. Sci. Rep..

[59] Sagi D (2011). Perceptual learning in Vision Research. Vis. Res..

[60] Verwey WB, Wright DL (2004). Effector-independent and effector-dependent learning in the discrete sequence production task. Psychol. Res..

[61] Verwey WB, Wright DL, Immink MA (2022). A multi-representation approach to the contextual interference effect: Effects of sequence length and practice. Psychol. Res..

[62] Van Duinen H, Gandevia SC (2011). Constraints for control of the human hand. J. Physiol..

[63] Ackerman PL (1987). Individual differences in skill learning: An integration of psychometric and information processing perspectives. Psychol. Bull..

[64] Chen G, Gully SM, Whiteman J-A, Kilcullen RN (2000). Examination of relationships among trait-like individual differences, state-like individual differences, and learning performance. J. Appl. Psychol..

[65] Tomassini V (2011). Structural and functional bases for individual differences in motor learning. Hum. Brain Mapp..

[66] Friedman J, Korman M (2012). Kinematic strategies underlying improvement in the acquisition of a sequential finger task with self-generated vs. cued repetition training. PLoS One.

[67] Voelcker-Rehage C (2008). Motor-skill learning in older adults—a review of studies on age-related differences. Eur. Rev. Aging Phys. Act..

[68] Censor N, Sagi D, Cohen LG (2012). Common mechanisms of human perceptual and motor learning. Nat. Rev. Neurosci..

