# Supplementary material for: Initial motor skill performance predicts future performance, but not learning
Source: Sci Rep. 2023 Jul 13;13:11359. doi: 10.1038/s41598-023-38231-5 (PMC10344907; doi:10.1038/s41598-023-38231-5)
Supplement: Supplementary file 1 — Supplementary Information. [file 41598_2023_38231_MOESM1_ESM.docx]

**Supplementary Information**

***Table s1***

*Extracted features and definitions for the machine learning pipeline*

| *Feature* | *Definition* | *Additional info* |
| --- | --- | --- |
| Start performance | Average of trials number 2 and 3 in a session | Accounting for warm-up decrements (Adams, 1952; Rickard et al., 2008). |
| End performance | Average of last 3 trials in a session |  |
| Maximal performance | Average of the 3 trials with highest performance in a session |  |
| Minimal performance | Average of the 3 trials with lowest performance in a session |  |
| Learning | Difference between the average of the 3 best trials in each session | Accounting for fatigue effects |
| Offline gains | Difference between the *Start performance* in session n+1 and the *End performance* in session n |  |
| Continuity | Average of the longest consecutive correct keypress of each trial across an entire session (Herszage et al., 2021) |  |
| Mean accuracy | Average accuracies of all trials within a session |  |
| Mean initial RT | Response time of the first keypress of each trial, averaged across a session | Used as an estimation of attentiveness |
| End of session slopes | A regression line (intercept and slope) fitted to the number of correct trials for the last 15 trials in a session | Slope in session 4 was fitted on all 9 trials |
| Micro on-line and off-line gains | Calculated for the first 5 trials in a session, based on Bönstrup et al., 2019 |  |
| **LOWESS (Locally Weighted Scatterplot Smoothing) based features** | The performance curve, using *statsmodel.aparametric.lowess* with default parameters (Seabold & Perktold, 2010) |  |
| Region of plateau | The longest streak of consecutive trials in which the derivative was below 0.25 | This located the longest streak of stable skill (no change in performance) |
| plateau start | The trial in which the plateau started |  |
| plateau end | The trial in which the plateau ended |  |
| streak count | plateau end - plateau start (in number of trials) |  |
| max smoothed performance | The maximum value of the smoothed curve |  |
| max smoothed index | the Index of the maximal value on the smoothed curve |  |
| **Within-sequence consistency dynamics** | The time difference between the first and last keypresses for each correct sequence |  |
| running RT consistency | The standard deviation of a running average (window of 10 sequences) was then extracted and fit with a 3^rd^ degree polynomial |  |
| RT consistency coefficients | the coefficients of a 3rd degree polynomial fitted on the running RT consistency data |  |
| RT consistency RMSE | Root mean squared error of the polynomial fit | A measure of how well the pattern consistency is captured by the polynomial |
| Pattern consistency trend | The Spearman correlation with the corresponding vector of window number within the session | Identify a stable trend in improvement of pattern execution times |
| **Session dynamics** | Learning curve – session performance:   1. $T_{n}= T_{1}n^{-l\left( n \right)}$ 2. $l\left( n \right)=l+ f_{p}+1-\exp\left( f_{p}\left( n^{f_{p}}-1 \right) \right)$ | Adapted from (Asadayoobi, Jaber, & Taghipour, 2021)  $l$ - learning rate  $f_{p}$ Fatigue parameter  $T_{n}$ – Performance in trial n  $n$ Trial number |

*Additional modeling approaches*

To assure that the main results are not due to a specific modeling family, we further examined different computational approaches, aimed at the same prediction targets, with different inductive biases as follows.

The first approach utilized the engineered features as predictors and examined a wide range of machine learning techniques. Specifically, we tested two tree-based models: Random Forest regression (Ho, 1995) and Sequential Regression Trees using gradient boosting (Xgboost; Chen & Guestrin, 2016), Regularized regression (Elastic net ;Zou & Hastie, 2005) and a multi-layer perceptron (MLP ;Haykin, 1994)). Due to the large number of potential predictors, and to avoid over-fitting of the training set, we tested these pipelines both with and without an additional preprocessing step of principle components analysis (PCA)-based dimensionality reduction. Each modeling pipeline started with a standard scaler, transforming the feature values into z-scores. We used grid search for hyper-parameters tuning of the algorithms and regularization parameters. Each set of hyper-parameters was optimized separately for each type of algorithm, predictors step and time interval. The best model was selected based on the average 5-fold cross validation (CV) score. For each model type and time interval, the model selection was done in stages. In each stage an additional set of predictors was introduced based on their complexity, starting with high level features (i.e., session dynamic parameters) and ending with the simplest features (performance per trial). Initially, only non-behavioral features were included (i.e., Age and Gender). Next, predictors were introduced in steps, consistent with the step employed in the main model reported in the study (see table s1). In the 1^st^ step parameters from the learning curve were introduced. The 2^nd^ step included the parameters extracted to capture *Within sequence consistency dynamics* and the *pattern consistency trend*. The 3^rd^ step included *Lowess based features*. The 4^th^ step included *session statistics*. The 5^th^ step included the micro-offline and micro-online features of the first 5 trials (Bönstrup et al., 2019). And the 6^th^ and final step, included the performance per trial for all trials in the session. For prediction purposes, normalization was done using the means and standard deviations of the variables in the training set. Additionally, we tested a recurrent Long Short-Term Memory (LSTM) network architecture in which the input was the number of correct sequences in all trials of the first session. This measure is the most common end-point measure in motor sequence learning studies (de Beukelaar, Woolley, & Wenderoth, 2014; Herszage & Censor, 2017; Herszage, Sharon, & Censor, 2021; Kami et al., 1995).

The second approach examined the prediction of future learning, based on all previous sessions. We used a linear regression model with correlation-based feature selection, introducing all available predictors at once and running a hyperparameters grid search on the number of selected features.

The applied models cover a wide array of approaches: Random Forest regression and Xgboost, the main models used in the study, use an ensemble of weak learners and aggregate their predictions either based on consensus (random forest regression) or in a sequential manner. A Multi-layered Perceptron (MLP), on the other hand, is a simple deep learning architecture consisting only of fully connected layers. The main advantage of these algorithms is their ability to capture interactions and other non-linear effects between predictors without explicitly modeling them by creating new variables. Regularized linear regression techniques were also examined due to their straightforward interpretability. Specifically, ElasticNet uses both L1 (Lasso) and L2 (Ridge) regularization penalties to limit model complexity while maintaining the linear relation between features and target.

Consistent with the main results, the models did not predict learning in the hold-out set (results for the best model across all modeling family types, per each interval. *session2 - session1*: *R^2^_mean_cv_score_*= 0.08, *R^2^_test_* =0.15; *session3 - session1*: *R^2^_mean_cv_score_*= 0.09, *R^2^_test_=-0.18; Retention session 4 - session1: R^2^_mean_cv_score_* = 0.01, *R^2^_test_* = 0.07, figure s1a). Similarly, the models did not predict the retention over the two retention intervals examined: (*Retention session - session3*: *R^2^_mean_cv_score_*= 0.11, *R^2^_test_ =* -0.84; *Long-retention – Retention session:*  *R^2^_mean_cv_score_*= 0.10 *R^2^_test_ =* -0.65*,* figure s1b)


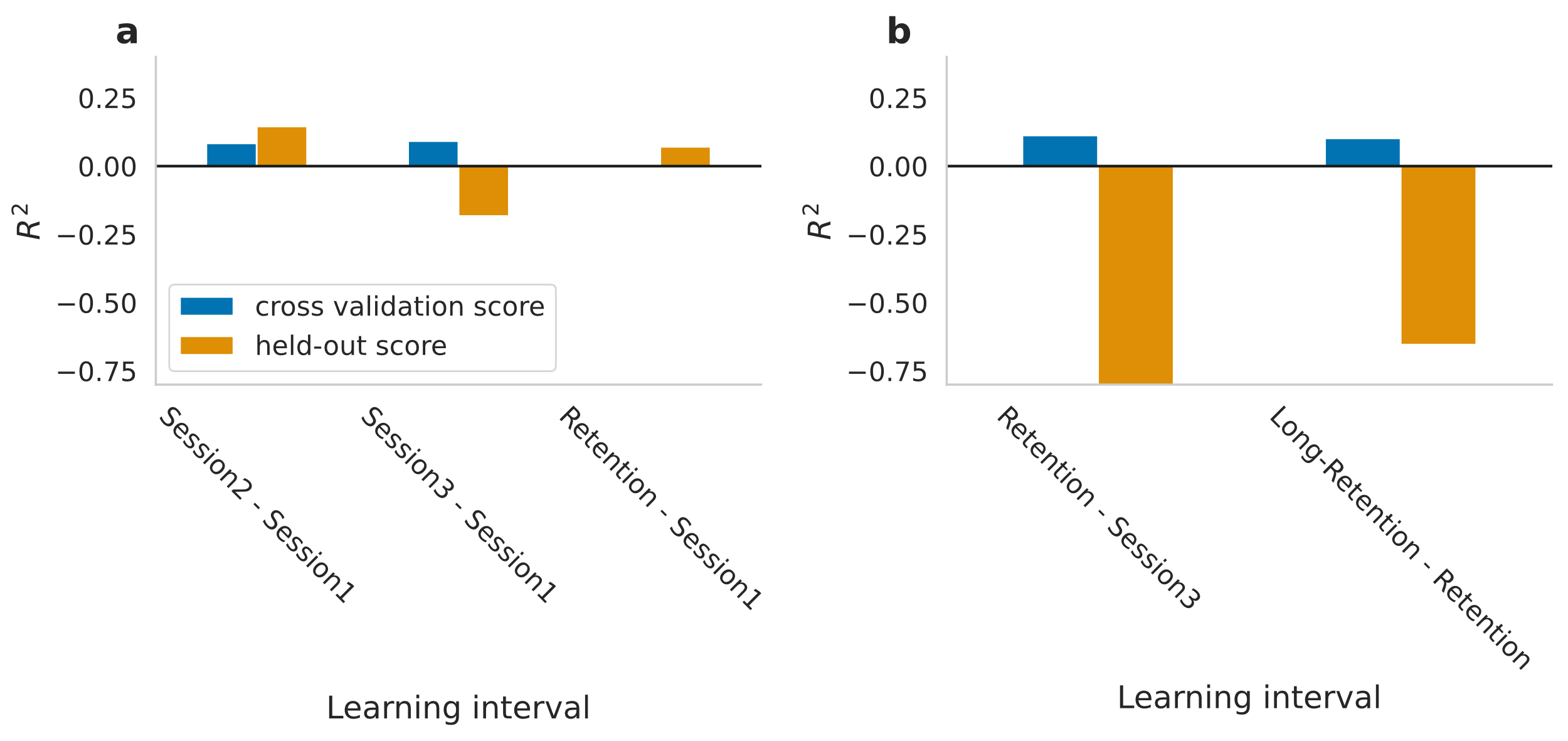


**Figure s1:** **Best model performance per interval with engineered features.** a) maximum mean cross-validation R^2^ scores (blue) and the corresponding hold-out R^2^ scores (orange) for each learning interval (X axis). b) Maximum mean cross-validation R^2^ scores (blue) and the corresponding hold out R^2^ (orange) for the two retention intervals (x axis).

In the third approach, models were trained directly on raw data from the first session, predicting learning between the first and second training sessions. Task performance was represented as a binary image of size 4 x 7200, where rows represent the key identity (1-4) and columns represent the time where the key was pressed (in 50ms bins). For example, a key press on the key “3” performed 250ms after trial start, will have a value of 1 in the coordinate (3,5). We then trained a convolutional neural network to predict learning. Hyper parameters of the topology and the optimization parameters were tuned manually. Similarly, a convolution encoder-decoder based method was built using the above binary session image as input, geared to reproduce the same image with a compact embedding layer which is then used as features in a regression analysis.

Next, we tested whether a different approach of machine learning models, avoiding feature selection based on prior assumptions, will achieve better prediction of future learning. To further investigate prediction in that direction, we trained a convolutional neuronal network on data from session 1, represented as a binary matrix of size 4 x 7200, where rows represent key identity and columns represent keypress time within the session in 50ms time bins (Figure s2). This representation reflects the available raw data, without imposing any definition of key correctness. This analysis was focused on the prediction of learning between the first and the second session, which includes the largest pool of participants. Additionally, to better utilize all available data, evaluation of model performance was based solely on cross validation. The best model resulted in mean cross validation *R^2^_test_*=-0.049, std = 0.053 performance. Consistent with this result, two additional models, using a convolution-based encoder-decoder and LSTM architectures (see above), did not show predictive power.


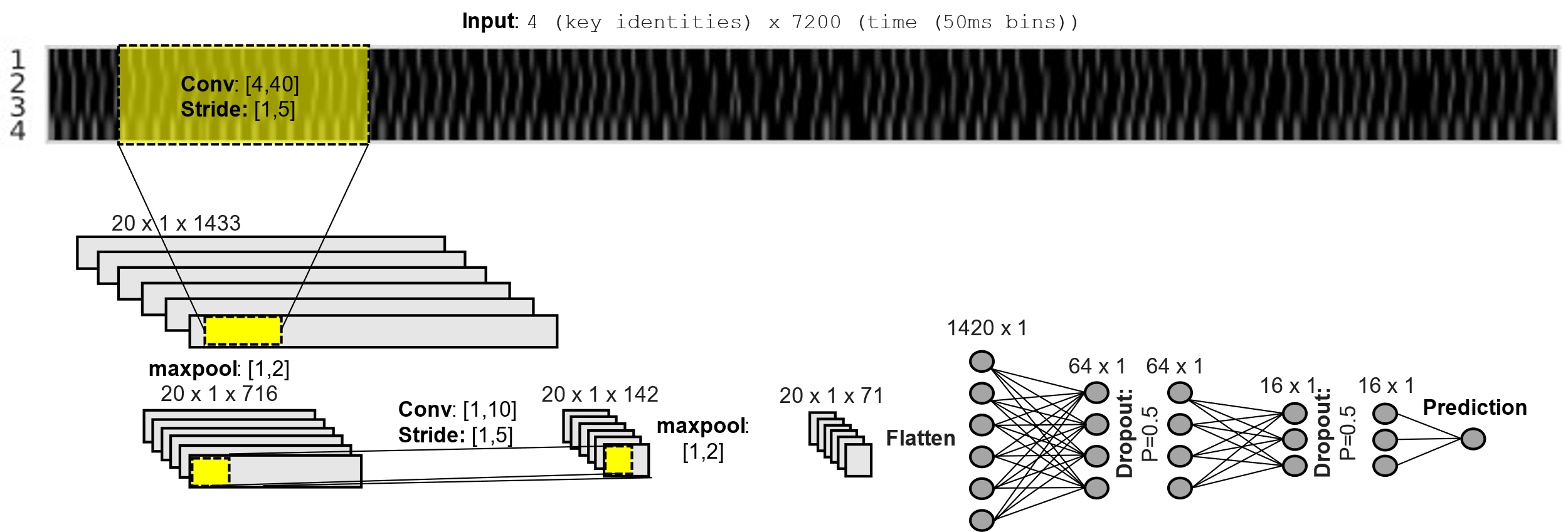


**Figure s2. Convolution based neural network architecture.** Input was represented as a 4 x 7200 binary matrix, where rows represent key identity (1-4) and columns represent time within the session (in 50ms time bins). The network architecture consists of two convolution layers, each followed by a pooling operation which is followed by 3 fully connected layers. The Rectified linear unit (Relu) was the selected activation function.
